# Single Nucleotide Polymorphism in the Promoter of the Human Interleukin-13 Gene Is Associated with Asthma in Malaysian Adults

**DOI:** 10.1155/2013/981012

**Published:** 2013-06-25

**Authors:** Ammu Kutty Radhakrishnan, Vijaya Lechimi Raj, Lee-Keng Tan, Chong-Kin Liam

**Affiliations:** ^1^Pathology Division, Faculty of Medicine and Health, International Medical University, 126, Jalan Jalil Perkasa 19, Bukit Jalil, 57000 Kuala Lumpur, Malaysia; ^2^Department of Pharmacology, Faculty of Medicine, MAHSA University, Jalan Elmu, Off Jalan University, 59100 Kuala Lumpur, Malaysia; ^3^Department of Medicine, Faculty of Medicine, University of Malaya, Lembah Pantai, 50603 Kuala Lumpur, Malaysia

## Abstract

Asthma susceptibility genes are mapped to a region on human chromosome 5q31-q33, which contains a cluster of proinflammatory cytokine genes such as interleukin-13 (IL-13), which is associated with asthma. This study investigated the allele frequencies of two single nucleotide polymorphisms (SNPs) (−1111C>T and 4257C>A) in the *IL-13* gene between asthmatics and healthy volunteers as well as the relationship between these SNPs and IL-13 production. DNA extracted from buffy coat of asthmatic and control subjects was genotyped using the PCR-RFLP method. Amount of IL-13 produced by mitogen-stimulated peripheral blood leucocytes PBLs (PBLs) was determined by ELISA. The frequencies of the −1111C and 4257G wild-type alleles were 0.52 and 0.55 in asthmatics and were 0.67 and 0.56 in controls. A significant (*P* < 0.05) association was found between genotype and allele frequencies of SNP at position −1111C>T between asthmatic and control groups (OR, 1.810; 95% CI = 1.184 to 2.767; *P* < 0.05). The mitogen-stimulated PBLs from asthmatics produced higher amounts of IL-13 production (*P* < 0.001). The 4257GA heterozygous and 4257AA homozygous mutant alleles were associated with higher IL-13 production in asthmatics (*P* < 0.05). Our results show that the −1111T mutant allele are associated with asthma and the 4257A mutant alleles are associated with elevated IL-13 production.

## 1. Introduction

Asthma is one of the most common respiratory disorders encountered in both children and adults. Over 300 million people worldwide suffer from asthma. Deaths due to asthma are estimated to increase by almost 20% in the next 10 years if urgent action is not taken. The international patterns of the prevalence of asthma are not explained by the current knowledge of the causes of asthma. Research into the causes of asthma and the efficacy of primary and secondary intervention strategies represent key priority areas in the field of asthma research [1]. Development of asthma is multifactorial and depends on interactions between multiple susceptibility genes and environmental factors [2–5]. According to the Expert Panel Report 2 from the National Heart, Lung and Blood Institute's (NHLBI) [6], the genetic predisposition for the development of an IgE-mediated response to common allergens is the strongest identifiable predisposing factor for developing asthma. High serum IgE levels have been reported to be correlated with the clinical expression of allergy and asthma, and a strong genetic component has been shown to contribute to this association [7, 8]. Recently, several whole-genome scans have been conducted to clarify the genetic basis of this complex disease. Genetic studies in several populations have identified a region on chromosome 5q31-q33 that contains the asthma susceptibility gene in several populations [1, 7–10]. Two members of this gene cluster, IL-4 and IL-13, have been shown to have a role in the pathogenesis of asthma [9, 10]. The *IL-13* gene (Locus ID 3596, NCBI, NIH) is closely linked to the *IL-4 gene*, and the IL-13 protein is reported to have a 30% identity in the amino acid sequence with the IL-4 protein [11]. Interleukin-13 induces its effects through a multisubunit receptor that includes a shared receptor composed of a heterodimer of IL-4R*α* and IL-13R*α*1 [12]. In addition, IL-13 and IL-4 elicit many of the same biologic responses without noticeable synergism [13]. This is because these cytokines have a common receptor component, that is, the alpha chain of the IL-4 receptor (IL-4R*α*) [14]. The relatively low affinity of IL-13 for IL-13R*α*1 is increased by approximately tenfold in the presence of IL-4R*α* [15]. The second IL-13 receptor, IL-13R*α*2, is not shared with IL-4 and is believed to be nonsignalling and may serve as a “decoy” receptor [16]. Both IL-13 and IL-4 are produced by T-helper-2 (T_H_2) cells in response to antigen presentation. The binding of IL-4 and IL-13 to IL-4R*α* triggers various cellular events that activate the transcription of germ-line epsilon (*ε*) heavy-chain gene locus. This event together with the signals derived from ligation of the B-cell surface molecule CD40, with its receptor will induce class switching in B-cells [17]. In humans, IL-13 levels are upregulated in asthmatics both systematically and in the lungs during asthmatic attacks [18]. Furthermore, IL-13 exhibits stimulatory activity to multiple cell types that are involved in asthma, including B cells, mast cells, eosinophils, pulmonary epithelial cells, fibroblasts and airway smooth muscle cells [19]. Several single nucleotide polymorphisms (SNPs) of the human *IL-13 gene* have been reported [1, 9]. Some of these SNPs are reported to have an effect on the complex regulation of asthma and atopy in the Dutch population. Howard and coworkers [1] showed that two of the SNPs (−1111C>T and 4257G>A) in the human *IL-13 gene* have the strongest relationship with the incidence of asthma in the Dutch population. This is a pilot study conducted on the Malaysian population to investigate the allelic frequency of two SNPs (−1111C>T and 4257G>A) in the human* IL-13 gene* and its effect on IL-13 production. This study can provide valuable insight into the overall mechanisms that cause susceptibility to asthma. As specific genes associated with clinical features of asthma are defined, the patterns developed may be useful in outlining important biologic pathways, leading to better understanding of this disease. The outcome of this study may provide a basis for future studies and contribute to early detection and diagnosis of asthma, as well as development of more effective therapeutic approaches. 

## 2. Materials and Methods

### 2.1. Volunteer Screening and Selection

Eighty-seven (87) patients with physician-diagnosed asthma and 94 normal subjects were recruited for this study from the outpatient clinics and medical wards of University Malaya Medical Centre (UMMC), Kuala Lumpur, Malaysia. The diagnosis of asthma was defined according to guidelines by the Global Initiative for Asthma (GINA) [20] (http://www.ginasthma.org/), and patients with all levels of asthma severity were recruited. Inclusion criteria include (i) age from 21 to 72 years; (ii) never smokers; (iii) no respiratory tract infections within 30 days prior to recruitment; (iv) no other significant medical illnesses which, in the opinion of the investigator(s), may either put the patient at risk because of participation in the study, influence the result of the study, or influence the patient's ability to participate in the study; (v) no conditions associated with alcohol or drug abuse; (vi) no participation in another clinical study of any investigational drug 30 days prior to or during this study; and (vii) agreement to give signed informed consent. Patients who did not fulfill these criteria were excluded from this study. Normal subjects were defined as having no respiratory diseases or symptoms, no allergies, and with normal lung function. The sample size was calculated using a formula provided by the website of St George's Hospital Medical School, Grant Application protocol (http://www-users.york.ac.uk/~mb55/guide/guide14.pdf). Wild-type and mutant allele frequencies chosen were based on estimation of the allele frequencies reported in the Japanese population [21].

### 2.2. Approval

The study and experiment protocol as well as collection of plasma and DNA materials were approved by the Ethics Committee of the University Malaya Medical Centre, Kuala Lumpur, Malaysia. 

### 2.3. Amplification of Genomic DNA by PCR

Five mL of venous blood was drawn from all study subjects and collected into heparinized tubes (Meus, Piove di Sacco Italy). The DNA was extracted from the buffy coat from 1 mL of blood using a DNA extraction kit as recommended by the manufacturer (Qiagen, Hilden, Germany). The SNP at position −1111C>T in the promoter region and 4257G>A in the coding region of the human IL-13 gene was amplified by PCR using published forward and reverse primers [22, 23]. The primers used are shown in Table 1. To perform PCR, AccuPower PCR PreMix tubes (BIONEER, Daejeon, Republic of Korea) preloaded with 1U of *Taq* DNA polymerase, 250 *μ*M dNTPs, 10 mM Tris-HCl (pH 9.0), 40 mM KCl, 1.5 mM MgCl_2_, stabilizer, and tracking dye were used. Total reaction volume was 20 *μ*L, and this contained 1 *μ*L of template DNA, 1 *μ*L of each primer (50 pmol/*μ*L), and 17 *μ*L ddH_2_0. Polymerase chain reaction was performed using a PTC-100 Peltier Thermal Cycler (MJ Research, St Waltham, MA, USA) using the following cycling conditions: 94°C for 5 minutes; 36 cycles at 94°C for 1 minute; 60°C for 1 minute, and 72°C for 1 minute, with a final extension step of 72°C for 5 minutes. The PCR products were checked using 1.5% agarose gel electrophoresis. The PCR products were purified using a commercial kit (GENOMED, Bad Oyenhausen, Germany). 

### 2.4. Restriction Fragment Length Polymorphism (RFLP) Analysis

About 2 *μ*L of the purified PCR product was then digested with appropriate restriction enzymes (Table 1). Digested samples were analysed by electrophoresis using 2% agarose gel for identification of SNP at site −1111C>T and 5% agarose gel for identification of SNP at site 4257G>A. 

### 2.5. Quantification of IL-13 Production by Peripheral Blood Leucocytes

Detection of IL-13 production by Concanavalin A- (Con A-) stimulated peripheral blood leucocytes (PBL) from the asthma patients and healthy volunteers was performed as described previously [24]. Briefly, 4 mL of blood from asthma patients and healthy volunteers was collected into heparin tube (Meus, Piove di Sacco, Italy). The blood was transferred into a 15 mL tube (Falcon, Becton-Dickinson, USA) and 8 mL of G-Dex II RBC lysis buffer (iNtRONS, Seongnam, Korea) was added to this tube. The mixture was vortexed and then centrifuged to recover the peripheral blood leucocytes (2,000 g at 4°C for 10 min). The pellet containing peripheral blood leucocytes was reconstituted with 5 mL complete RPMI 1640 medium (Gibco, Invitrogen, Carlsbad, CA, USA) supplemented with L-glutamine, 10% (v/v) fetal bovine serum (FBS), 100 units/mL penicillin, and 0.1 mg/mL streptomycin (Gibco, Invitrogen, Carlsbad, CA, USA). Cell count was adjusted to 2.5 × 10^5^ cells/mL with complete RPMI medium and 100 *μ*g/mL Concanavalin A (Con A) (Sigma-Aldrich, St. Louis, MO, USA). Then 200 *μ*L of this cell suspension was plated out in two sterile 96-well flat-bottom plates (Falcon, Becton Dickinson, USA). Culture plates and their lids were sealed with surgical tape (3 M) and incubated at 37°C in a humidified 5% CO_2_ incubator (SHELLAB, Cornelius, OR, USA). The plates were removed after 72 hours and stored at −80°C until further analysis. The PBLs were cultured for 72 hours in the presence of Con A as preliminary studies showed that IL-13 production reached significant levels only after 72 hours of culture (data not shown). The amount of IL-13 produced by the peripheral blood leucocytes was measured by commercially available enzyme-linked immunosorbent assay (ELISA) human IL-10 kit, according to the manufacturer's recommendation (eBioscience, San Diego, CA, USA). 

### 2.6. Statistical Analysis

Data was first tabulated in Microsoft Excel and transferred into SPSS (version 11.5, Chicago, IL, USA) and MINITAB (version 14, PA, USA) statistical packages for statistical analysis. Statistical differences between genotype and allele frequencies were compared using Chi-square test (*χ* ^2^-test) in MINITAB. Chi-square test was performed to determine if the association between genotype and allele frequencies of patients and control subjects was statistically significant. Allele and genotype frequency data was also subjected to the Hardy-Weinberg equilibrium test (data not shown). The Mann-Whitney test was used to compare IL-13 production by Con A-stimulated peripheral blood leucocytes between asthmatic and control groups. 

## 3. Results

### 3.1. Genotype and Allele Frequency Analysis

**Figure 1:**
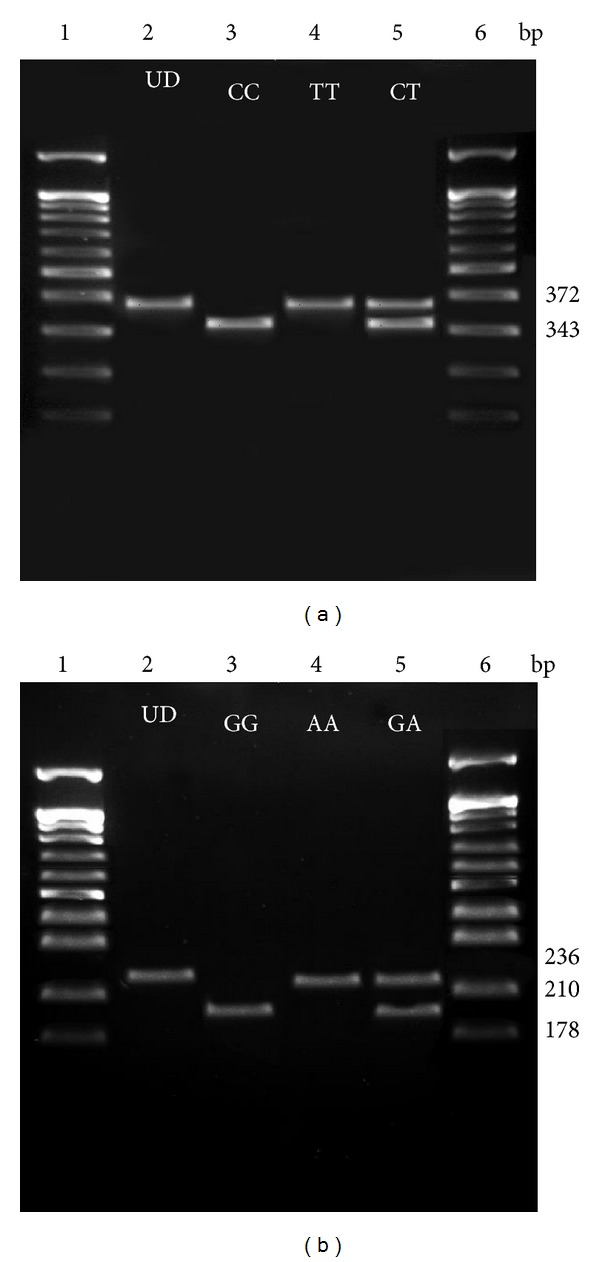
Gels representing the typical restriction fragment length polymorphism (RFLP) patterns obtained following digestion with the restriction enzymes (a) *Hpy99I* and (b) *Nla*IV, respectively. The restriction enzyme (a) *Hpy99I* recognises an SNP in the promoter region at site −1111C>T while *Nla*IV recognises an SNP in the coding region at position 4257G>A. In both gels, *Lanes 1* and *6* represent 100 bp DNA ladder (New England Biolabs, Beverly, MA, USA); *Lane 2* represents undigested PCR amplified products (UD) while *Lane 3* represents the typical RFLP pattern seen when only the respective homozygous wild-type alleles are present; *Lane 4* shows the typical RFLP pattern seen when the only respective homozygous mutant alleles are present while *Lane 5* shows the RFLP pattern seen when the respective heterozygous alleles are present.

Presence or absence of SNP at positions −1111C>T and 4257G>A was detected by PCR-RFLP methods. Typical restriction patterns obtaining following digestion of purified PCR products are shown in Figure 1. Results from PCR-RFLP methods were validated with DNA sequencing (data not shown). Genotype data obtained from RFLP was first tested with Hardy-Weinberg equilibrium (HWE) test before further genetic analysis. There was no significant deviation of the HWE observed in either asthmatic or control groups (results not shown here). A majority of the patients and control subjects included in this study were found to be heterozygous (CT) at position −1111C>T (Table 2(a)). However, most control subjects were found to have two wild-type alleles (CC) at this site compared to the asthmatic patients (Table 2(a)). For SNP at position 4257G>A, most subjects were found to be heterozygous (GA) and carrying the wild-type G allele. A higher percentage of asthmatic patients were found to carry homozygous wild-type (GG) and homozygous mutant (AA) compared to control subjects (Table 2(a)). 

### 3.2. Allelic Frequencies

At position −1111C>T, a higher percentage of asthmatic and control subjects were found to carry the wild-type C allele compared to the mutant T allele (Table 2(b)). However, more control subjects were found to have the wild-type allele compared to asthmatic patients. Asthmatic patients were found to possess a higher percentage of mutant T allele compared to control subjects. The difference in allele frequency of SNP at site −1111C>T between asthmatic and control groups was found to be statistically significant (*P* < 0.006). However, the difference in allele frequency of SNP at site 4257G>A between asthmatic and control groups was not statistically significant (Table 2(b)). 

### 3.3. Association Studies

The association between genotype and allele frequencies with patient and control groups was found to be significant for the SNP at site −1111C>T (*P* < 0.05). Hence, the odds ratio of the two alleles (C and A) at site −1111C>T occurring in both groups was calculated. The odds ratio of 1.810 was found to be significant with 95% confidence interval (1.184 to 2.767) (Table 3). There was no significant association found between genotype and allele frequencies with patient and control groups for SNP at site 4257G>A (*P* > 0.05).

### 3.4. Relationship between SNP in IL-13 Gene and IL-13 Production

**Figure 2:**
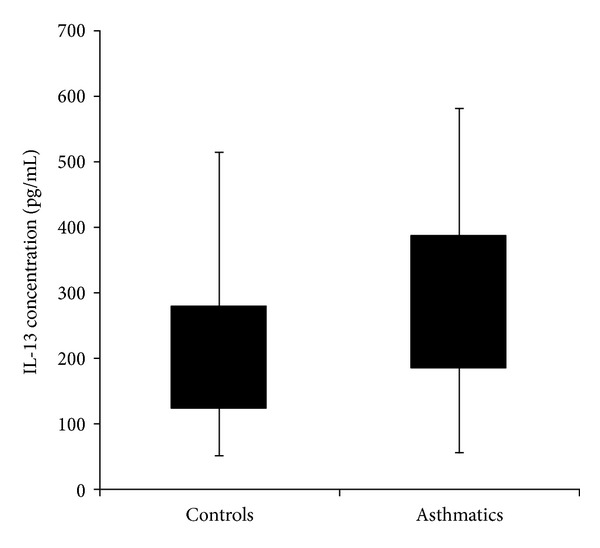
Boxplot showing IL-13 production by Con A-stimulated PBL from asthmatic patients and control subjects. Mean productions of IL-13 by the Con A stimulated from asthmatic patients and control subjects were 282.11 ± 120 and 209.21 ± 99, respectively. Production of IL-13 by Con A-stimulated PBL from asthmatic patients was significantly (*P* < 0.001; Mann-Whitney *U* Test) higher compared to controls.

The amount of IL-13 produced by Con A-stimulated peripheral blood leucocytes (PBL) was analysed after 72 hours of culture. The PBL from asthmatics produced significantly (*P* < 0.001) higher amounts of IL-13 compared to controls (Figure 2). Asthmatic patients carrying the homozygous wild-type or mutant allele at position −1111C>T produced significantly (*P* < 0.001) higher amounts of IL-13 compared to control subjects with the same genotypes (Table 4). In contrast, asthmatic patients carrying the heterozygous (*P* < 0.011) or homozygous (*P* < 0.001) mutant allele at site 4257G>A produced significantly higher amounts of IL-13 compared to control subjects with the same genotypes (Table 4). 

## 4. Discussion

The characterisation and identification of genes influencing complex disease and traits are a major goal of human genetics. However, the mapping of complex disease gene may be complicated when there is heterogeneity in the aetiology of the disease, as in asthma. In this study, a significant association was found between genotype and allele frequencies of the SNP in the promoter region (−1111C>T) with asthmatic patients and control subjects (Table 2). These results are consistent with previous studies done on the Dutch [1, 8] and African American populations [22]. Individuals with mutant T-allele at position −1111C>T of the human IL-13 gene promoter have a 1.81 times likelihood to develop asthma compared to those without this allele at this SNP. This suggests that the mutant T-allele at position −1111C>T of the human IL-13 gene promoter may have an effect on the development of asthma in Malaysian asthmatics. For the SNP at site 4257G>A on the IL-13 gene, the homozygous mutant alleles (AA) and the mutant-A allele were found to be more prevalent in asthmatic patients compared to control subjects. However, no significant association between genotype and allele frequencies between this SNP and asthma was found. These results matched the findings from similar studies done on the Dutch [1], Chinese [23], and Mexican [25] populations but differed from studies done on the British, Japanese [21, 26], and Korean [27, 28] populations. Association analyses with the polymorphism at site 4257 in the IL-13 gene produced varied results [1, 8, 21, 26, 27]. This may be partly due to the fact that each study is based on population samples that were ascertained differently. Another potential cause of variability in the results of these studies is that they were performed in different population groups. It is also possible that different polymorphisms within the IL-13 gene may contribute to the development of asthma in different populations. Therefore, each analysis may be identifying the specific allele responsible for the phenotype in a specific population. This suggests that different polymorphisms identified are capable of altering the function of IL-13 and thus contributing to a predisposition to asthma. 

The allele frequencies for this study showed that there was a high frequency of the C-allele as compared to the T-allele at position −1111C<T of the human IL-13 gene promoter in both asthmatics and control subjects. The frequency for the T-allele at the same position was relatively lower in both asthmatics and control subjects. This frequency differed from that reported for the Dutch [1] and Korean [27, 28] populations studies where the frequency of the −1111C allele was reported to be higher in both asthmatics and control subjects. However, the frequencies for the C- and T-alleles in this site were similar to that reported in the African American population [22]. For the SNP at position 4257G>A of the human IL-13 gene, allele frequencies for the G-allele in Malaysian asthmatics and control subjects recruited in this study were found to be similar to the frequencies reported for the Koreans [28] and the Mexicans [25]. In comparison to the Malaysians, the Dutch population reported a higher frequency for the 4257G allele compared to the 4257A allele in both asthmatics and control subjects [1]. In contrast, the same allele frequency was found to be lower in Japanese asthmatics [26] and Chinese controls [23] compared to the Malaysians. However, frequencies for the control group in the Japanese study were similar to the controls in this study [26]. Comparison of allele frequencies with previous studies showed that there were significant interpopulation differences in allele frequencies of the IL-13 polymorphisms. The variations observed in the different populations were most likely due to current concepts of asthma pathogenesis that the onset of disease and its clinical course are determined by the interaction between genetic and environmental factors; that is, those people in whom asthma develops are genetically susceptible and receive an appropriate environmental stimulus. Thus, IL-13 polymorphisms may have varied and population-specific effects.

There was a significantly higher production of IL-13 by the Con A-stimulated PBL isolated from the asthmatics compared to control subjects. This finding further supports the hypothesis that IL-13 plays an important role in the pathogenesis of asthma [29]. The relationship between the presence of functionally relevant polymorphisms in the IL-13 gene and IL-13 production is of clinical interest because of the regulatory role of IL-13 in the pathogenesis of asthma along with interindividual differences in IL-13 production capacity [1, 10, 11]. It was observed from the data that asthma patients carrying the −1111CC homozygous wild-type alleles (*P* < 0.001) and −1111TT homozygous mutant alleles produced significantly higher IL-13 compared to control subjects carrying the same genotypes. This suggests that homozygous alleles in the promoter region (−1111C>T) of the Malaysian asthmatics may contribute to higher IL-13 production. A previous study found that the −1111TT genotype is associated with significantly altered regulation of IL-13 production in the Dutch population [8]. 

A significantly higher mean for IL-13 production was found in asthmatics carrying the 4257GA heterozygous alleles and 4257AA homozygous mutant alleles compared to control subjects who carried the same genotypes. These results suggest that the mutant A-allele might have an effect on IL-13 production in the Malaysian asthmatics. A similar finding was reported in the Japanese population [26]. Previous modelling studies have showed that the SNP at site 4257G>A occurs in a site crucial for ligand-receptor interactions resulting in an IL-13 protein with enhanced binding affinity for its receptor [26]. Furthermore, it was proposed that this polymorphism could also alter the stability of the IL-13 messaging system and/or the metabolism of the IL-13 proteins leading to increased production [11]. 

From this study, the relationships of the profile of the two SNPs (−1111C>T and 4257G>A) were established with gender and ethnicity. There was no significant association found between genotype and allele frequencies of the SNPs in the two regions with gender and the three major ethnic groups in Malaysia. The allele frequencies in asthmatics and control subjects were compared with allele frequencies reported in other populations and significant interpopulation differences in allele frequencies of IL-13 polymorphisms were found.

This study showed significantly higher IL-13 production in asthmatic patients compared to control subjects, and this supports the idea that IL-13 plays an important role in the pathogenesis of asthma. The association studies between the SNPs with IL-13 production in asthmatics and control subjects indicated that SNP at site −1111C>T resulted in patients carrying homozygous wild-type CC and homozygous mutant AA alleles and having higher IL-13 production. The results also showed that the 4257A mutant allele is associated with significantly higher IL-13 production in asthmatics compared with control subjects.

## Figures and Tables

**Table 1:** Technical data for the analysis of single nucleotide polymorphisms (SNPs) in the human *IL-13 gene*.

Region	Primers		PCR product size (bp)	Restriction enzyme*	Annealing temperature	Reference
Promoter: (**−1111C>T**)	Forward: Reverse:	5′-ACT TCT GGG AGT CAG AGC CA-3′ 5′-TAC AGC CAT GTC GCC TTT TCC TGC TCT TCC GTC-3′	372	*Hpy99I *	60°C	[22]

Coding: (**4257G>A**)	Forward: Reverse:	5′-CTT CCG TGA GGA CTG AAT GAG ACG GTC-3′ 5′-GCA AAT AAT GAT GCT TTC GAA GTT TCA GTG GA-3′	236	*Nla*IV	60°C	[23]

GenBank accession number U31120 was used as the reference sequence.

*New England Biolabs, Beverly, MA, USA.

**Table:** (a)

Site	Genotype	Frequencies		*P* value^a^	DF^b^
		Asthmatic patients (*n* = 87)	Control subjects (*n* = 94)		
	**CC**	20 (2.3%)	41 (43.6%)		
−1111C>T	**CT**	51 (58.6%)	43 (45.7%)	0.011	2
	**TT**	16 (18.4%)	10 (10.6%)		

	**GG**	28 (32.2%)	24 (25.5%)		
4257G>A	**GA**	39 (44.8%)	57 (60.6%)	0.086	2
	**AA**	20 (23.0%)	13 (13.8%)		

**Table:** (b)

Site	Genotype	Frequencies		*P* value^a^	DF^b^
		Asthmatic patients (*n* = 87)	Normal subjects (*n* = 94)		
−1111C>T	**C**	91 (52.3%)	125 (66.5%)	0.006	1
	**T**	83 (47.7%)	63 (33.5%)		

4257G>A	**G**	95 (54.6%)	105 (55.9%)	0.811	1
	**A**	79 (45.4%)	83 (44.1%)		

^a^Chi-square test.

^b^DF: degree of freedom.

**Table 3:** Odds ratio for −1111C>T SNP between asthmatic patients and control subjects.

Position	Allele	Asthmatic patients	Control subjects	Total
−1111	Mutant allele (**T**)	83	63	146
−1111	Wild-type allele (**C**)	91	125	216

	Total	174	188	

Odds ratio = 1.810.

95% confidence interval = 1.184 to 2.767.

**Table 4:** Relationship between the SNP in the human Il-13 gene and production of IL-13 by the Con A-stimulated peripheral blood leucocytes.

SNP	Genotype	Production of IL-13 (pg/mL)		*P* value^a^
		Asthmatic patients (mean ± SD)	Control subjects (mean ± SD)	
**−1111C>T**	**CC**	345.41 ± 117.71	222.63 ± 93.30	**<0.001**
	**CT**	252.96 ± 119.40	208.31 ± 107.64	0.089
	**TT**	295.92 ± 95.70	158.05 ± 65.40	**<0.001**

**4257G>A**	**GG**	264.16 ± 93.50	209.44 ± 91.54	0.068
	**GA**	285.62 ± 93.50	215.25 ± 103.46	**0.011**
	**AA**	300.41 ± 116.75	182.26 ± 92.72	**0.008**

^a^Mann-Whitney test.

SD: standard deviation.

## References

[1] Howard TD, Whittaker PA, Zaiman AL (2001). Identification and association of polymorphisms in the interleukin-13 gene with asthma and atopy in a dutch population. *American Journal of Respiratory Cell and Molecular Biology*.

[2] Cookson W (1999). The alliance of genes and environment in asthma and allergy. *Nature*.

[3] Holgate ST (1999). Genetic and environmental interaction in allergy and asthma. *The Journal of Allergy and Clinical Immunology*.

[4] Masoli M, Fabian D, Holt S, Beasley R (2004). The global burden of asthma: executive summary of the GINA dissemination committee report. *Allergy*.

[5] Ober C, Moffatt MF (2000). Contributing factors to the pathobiology: the genetics of asthma. *Clinics in Chest Medicine*.

[6] National Heart, Lung, and Blood Institute (NHLBI) (1997). Expert panel report 2. Guidelines for the diagnosis and management of asthma: national asthma education and prevention program. *The Journal of Allergy Clinical Immunology*.

[7] Van Der Pouw Kraan TCTM, Van Veen A, Boeije LCM (1999). An IL-13 promoter polymorphism associated with increased risk of allergic asthma. *Genes and Immunity*.

[8] Burrows B, Marinez FD, Halonen M, Barbee RA, Cline MG (1989). Association of asthma with serum IgE levels and skin-test reactivity to allergens. *The New England Journal of Medicine*.

[9] Graves PE, Kabesch M, Halonen M (2000). A cluster of seven tightly linked polymorphisms in the IL-13 gene is associated with total serum IgE levels in three populations of white children. *The Journal of Allergy and Clinical Immunology*.

[10] Wills-Karp M, Luyimbazi J, Xu X (1998). Interleukin-13: central mediator of allergic asthma. *Science*.

[11] Obiri NI, Leland P, Murata T, Debinski W, Puri RK (1997). The IL-13 receptor structure differs on various cell types and may share more than one component with IL-4 receptor. *The Journal of Immunology*.

[12] Chomarat P, Banchereau J (1998). Interleukin-4 and interleukin-13: their similarities and discrepancies. *International Reviews of Immunology*.

[13] Zurawski SM, Chomarat P, Djossou O (1995). The primary binding subunit of the human interleukin-4 receptor is also a component of the interleukin-13 receptor. *The Journal of Biological Chemistry*.

[14] Zurawski G, De Vries JE (1994). Interleukin 13 elicits a subset of the activities of its close relative interleukin 4. *Stem Cells*.

[15] Aman MJ, Tayebi N, Obiri NI, Puri RK, Modi WS, Leonard WJ (1996). cDNA cloning and characterization of the human interleukin 13 receptor *α* chain. *The Journal of Biological Chemistry*.

[16] Zhang J-G, Hilton DJ, Willson TA (1997). Identification, purification, and characterization of a soluble interleukin (IL)-13-binding protein. *The Journal of Biological Chemistry*.

[17] Bacharier LB, Geha RS (2000). Molecular mechanisms of IgE regulation. *The Journal of Allergy and Clinical Immunology*.

[18] Prieto J, Lensmar C, Roquet A (2000). Increased interleukin-13 mRNA expression in bronchoalveolar lavage cells of atopic patients with mild asthma after repeated low-dose allergen provocations. *Respiratory Medicine*.

[19] Vercelli D (2002). Genetics of IL-13 and functional relevance of IL-13 variants. *Current Opinion in Allergy and Clinical Immunology*.

[21] Hiromatsu Y, Fukutani T, Ichimura M (2005). Interleukin-13 gene polymorphisms confer the susceptibility of Japanese populations to Graves’ disease. *The Journal of Clinical Endocrinology and Metabolism*.

[22] Moissidis I, Chinoy B, Yanamandra K (2005). Association of IL-13, RANTES, and leukotriene C4 synthase gene promoter polymorphisms with asthma and/or atopy in African Americans. *Genetics in Medicine*.

[23] Leung TF, Tang NLS, Chan IHS, Li AM, Ha G, Lam CWK (2001). A polymorphism in the coding region of interleukin-13 gene is associated with atopy but not asthma in Chinese children. *Clinical and Experimental Allergy*.

[24] Hee CS, Gun SC, Naidu R, Gupta E, Somnath SD, Radhakrishnan AK (2007). Comparison of single nucleotide polymorphisms in the human interleukin-10 gene promoter between rheumatoid arthritis patients and normal subjects in Malaysia. *Modern Rheumatology*.

[25] López KIM, Martínez SEF, Moguel MCM (2007). Genetic diversity of the IL-4, IL-4 receptor and IL-13 loci in mestizos in the general population and in patients with asthma from three subpopulations in Mexico. *International Journal of Immunogenetics*.

[26] Heinzmann A, Mao XQ, Akaiwa M (2000). Genetic variants of IL-13 signalling and human asthma and atopy. *Human Molecular Genetics*.

[27] Kim JJ, Min JY, Lee JH (2007). Polymorphisms in the IL-13 and IL-4 receptor alpha genes and allergic rhinitis. *European Archives of Oto-Rhino-Laryngology*.

[28] Kim H-B, Lee Y-C, Lee S-Y (2006). Gene-gene interaction between IL-13 and IL-13R*α*1 is associated with total IgE in Korean children with atopic asthma. *Journal of Human Genetics*.

[29] Elias JA, Lee CG, Zheng T, Ma B, Homer RJ, Zhu Z (2003). New insights into the pathogenesis of asthma. *The Journal of Clinical Investigation*.

